# Public coverage of Long-Term Care in the post-COVID period: strengthening systems vs cost-containment

**DOI:** 10.1093/ppar/prag006

**Published:** 2026-05-14

**Authors:** Adelina Comas-Herrera, Nazak Salehi, Alexander Chaverri-Carvajal, Mari Aaltonen, Başak Akkan, Simon Bottery, Jorge Browne, Maria Cheshire-Allen, Margaret Dunham, Moira Dunsmore, Cengiz Erdoğan Cansu, Ulrike Famira-Mühlberger, Maria Aurora Fenech, Leena Forma, Maya Fransz-Myers, Vlad Grigoras, Ester Gubert, Ali Hamandi, Valentina Hlebec, Maša Filipovič Hrast, Lennarth Johansson, Marios Kantaris, Hongsoo Kim, David Knapp, Henk Nies, Eleonora Perobelli, Ruru Ping, Tjaša Potočnik, Jayeeta Rajagopalan, Roberta Sultana, Loïc Trabut, Cristina Vilaplana-Prieto, Pablo Villalobos Dintrans, Diego Wachs, Karen Watson, Raphael Wittenberg, Joseba Zalakain

**Affiliations:** Care Policy and Evaluation Centre, London School of Economics and Political Science, London, United Kingdom; Care Policy and Evaluation Centre, London School of Economics and Political Science, London, United Kingdom; Centre d’Estudis Demogràfics, Universitat Autònoma de Barcelona, Bellaterra, Spain; Department of Public Health and Welfare, Finnish Institute for Health and Welfare (THL), Helsinki, Finland; International Relations Department, Istanbul Bilgi University, Istanbul, Türkiye; Policy Directorate, The King’s Fund, London, United Kingdom; Department of Public Health, Pontificia Universidad Católica de Chile, Santiago, Chile; School of Health and Social Care, Swansea University, Swansea, Wales, United Kingdom; School of Health and Social Care, Edinburgh Napier University, Edinburgh, Scotland, United Kingdom; Sydney Nursing School, The University of Sydney, Sydney, New South Wales, Australia; Faculty of Sociology, Bielefeld University, Bielefeld, North Rhine-Westphalia, Germany; Health Economics and Health Policy Unit, Austrian Institute of Economic Research (WIFO), Vienna, Austria; Department of Health Services Management, University of Malta, Msida, Malta; Unit of Health Sciences, Tampere University, Tampere, Finland; Department of Health and Social Management, University of Eastern Finland, Kuopio, Finland; Center for Economic and Social Research, University of Southern California, Los Angeles, California, USA; Social Protection and Jobs Global Practice, World Bank, Washington, DC, USA; Department of Sociology and Social Research, University of Trento, Trento, Italy; Health, Nutrition, and Population Global Practice, World Bank, Washington, DC, USA; Faculty of Social Sciences, University of Ljubljana, Ljubljana, Slovenia; Faculty of Social Sciences, University of Ljubljana, Ljubljana, Slovenia; School of Health and Welfare, Jönköping University, Jönköping, Sweden; Health Services and Social Policy Research Centre, Cyprus University of Technology, Limassol, Cyprus; Department of Public Health Sciences, Graduate School of Public Health & Artificial Intelligence Institute, Seoul National University, Seoul, South Korea; Center for Economic and Social Research, University of Southern California, Los Angeles, California, USA; Department of Governance and Management in the Health and Care Sector, Vrije Universiteit Amsterdam, Amsterdam, Netherlands; CERGAS, SDA Bocconi School of Management, Bocconi University, Milan, Italy; Hitotsubashi Institute for Advanced Study, Hitotsubashi University, Tokyo, Japan; Faculty of Social Sciences, University of Ljubljana, Ljubljana, Slovenia; Care Policy and Evaluation Centre, London School of Economics and Political Science, London, United Kingdom; Department of Health Services Management, University of Malta, Msida, Malta; Mobility, Trajectories and territories Unit, French National Institut for Demographic Studies (Ined), Aubervilliers, France; Faculty of Economics and Business, University of Murcia, Murcia, Spain; 1. Escuela de Salud Pública, Facultad de Medicina y Ciencias de la Salud, Universidad Mayor, Santiago, Chile 2. Centro de Observación y Análisis de Datos en Salud (CADS), Universidad Mayor, Santiago, Chile; Social Protection and Jobs Global Practice, World Bank, Washington, DC, USA; Sydney Nursing School, The University of Sydney, Sydney, New South Wales, Australia; Care Policy and Evaluation Centre, London School of Economics and Political Science, London, United Kingdom; SIIS Research Centre, Fundación Eguía-Careaga, Donostia-San Sebastián, Basque Country, Spain

**Keywords:** Long-Term Care Systems, Universal Health Coverage, Long-Term Care Policy

## Introduction

In the context of population ageing all over the world, and improved life expectancy for people living with chronic conditions and disabilities, the need for Long-Term Care (LTC) is expected to grow globally, requiring LTC systems that offer sustainable and equitable responses (Carrino et al., 2025).

While the goal of Universal Health Coverage (UHC) has received significant attention in international health policy, with its own Sustainable Development Goal indicators and dedicated tracking (The World Health Organization and The World Bank Group, 2023), the concept of Universal Coverage of LTC to ensure coverage of the risks posed by needing care, has not been given similar attention. Relatively few countries have established comprehensive public coverage of the risk of needing LTC, with wide variation in the degrees of formalisation of care and of public coverage. There are also important differences in the boundaries of what constitutes the public LTC system as opposed to the health, pensions or social protection systems and, in many countries, there are multiple LTC schemes, with different coverage rules.

To advance understanding of the public coverage of the risk of needing LTC, we review mechanisms and policies that countries have in place (or not) to determine LTC coverage, building on the framework provided by World Health Organization’s UHC (Chisholm et al., 2010), with three key questions to determine the extent and depth of a system’s coverage: who is covered, what is covered, and how much is covered.

We also explore whether there is a trend towards improving coverage of the risk of needing LTC, or whether policy progress on LTC coverage is stagnating or even retreating in the context of competing priorities for public spending. We focus on the post-COVID period (since 2021), when increased public and political awareness of the need to strengthen LTC systems (World Health Organization, 2020) may have increased support and momentum for LTC reforms, exemplified in Europe and Latin-America with the adoption of the European Care Strategy (Daly, 2025, (Council of the European Union, 2022) and PAHO/WHO’s launched Long-Term Care Policy (PAHO/WHO, 2024).

On the other hand, the COVID-19 pandemic has been just one of many shocks affecting the global economy in recent years, leading to governments reordering their policy priorities (International Monetary Fund, 2025). This challenging economic and political climate could result in a lack of political appetite for reforms to LTC systems that may increase public expenditures or the adoption of cost-containment policies.

In the first part of this paper we discuss how 19 countries’ LTC systems cover people for the risk of needing LTC using the three questions posed by the WHO Universal Coverage framework (people, services and costs). We do not aim to describe all the policies in detail or provide quantitative estimates of the extent of coverage (see (Llena-Nozal et al., 2025), instead we focus on policy decisions that determine the extent and depth of coverage of public LTC systems.

Second, we explore whether these countries have adopted policies that expand the coverage of the LTC system, or if, instead, they have adopted cost-containment policies. We use a framework that identifies cost-containment demand and supply side policies (Gori & Luppi, 2024). We do not attribute the policy changes to the COVID pandemic but rather consider this as a period from when changes may have been expected.

We use the World Health Organization definition of LTC as “Activities to ensure that people with or at risk of a significant ongoing loss of intrinsic capacity can maintain a level of functional ability consistent with their basic rights, fundamental freedoms and human dignity”(World Health Organization, 2015). This includes all forms of care: unpaid care provided by family members, formal home- and community-based care, and residential settings such as assisted living facilities and nursing homes (World Health Organization, 2021).

## Methods

Building on the WHO UHC cube questions and Gori and Luppi’s (2024) cost-containment framework, we developed a questionnaire which was shared with experts on LTC systems from the Global Observatory of Long-Term Care network provided answers to a questionnaire (see appendix), describing the situation in August to September 2025.

The answers were summarised and collated into tables. These were shared with the country experts to ensure that the answers had been adequately understood and described. Based on their answers some additional coverage mechanisms were identified in a second iteration and added to the tables.

In terms of expansion versus cost-containment policies, the questions considered, on the demand side, policies that tightened or expanded eligibility criteria, policies that reduced or increased care responsiveness by influencing waiting times, and policies that increased or decreased the stringency of means-tests. On the supply side, the questions asked about changes in the mix of services available, the intensity and quality of services. As in many countries LTC coverage varies substantially by region, state, and localities, experts were asked to consider the main approaches in their country, noting where there was regional or local variability.

## Findings

We obtained findings for 19 diverse countries: Australia, Austria, Chile, Costa Rica, Croatia, Cyprus, Finland, France, India, Italy, Japan, Malta, the Netherlands, Republic of Korea, Slovenia, Spain, Sweden, Türkiye, the United Kingdom (separately England, Scotland, and Wales given substantial system differences), and the United States of America. Most are high-income economies (except for India and Türkiye), spanning five continents (Asia, Europe, North America, Oceania, and South America). The findings are presented for each of the Universal Coverage Cube questions, and then for the cost-containment framework.

### Part One: policies that determine LTC coverage

#### WHO is covered?

Most countries cover all nationals and people with residency permits and recognised asylum status provided they meet the eligibility criteria. In social insurance systems such as Japan, Republic of Korea, and Slovenia, people need to be enrolled in the LTC insurance system, with a temporal requirement in the case of Slovenia (having been insured 24 out of 36 months before application).

Regarding age, slightly more countries have a single system covering people of all ages instead of separate systems for older and younger adults with disabilities. The UK systems cover children with disabilities separately. Interestingly, Slovenia’s new social LTC insurance system is the same for all adults, unlike the other social insurance systems in the study which have parallel systems for younger and older people (Japan and Korea).

A key coverage distinction is whether a system covers all people with LTC-related needs (universal access), or only those who are poorer (means-tested access). 11 countries have universal systems, but all have co-payments, which we discuss later. Three countries offer at least some universal benefits, and six only offer publicly funded LTC support to those with income (and/or assets) below a means-test (see table 1).

**Table 1:** Universal versus means-tested access to public Long-Term Care support.

Access to the main LTC public scheme is universal, based only on need^a^	Main LTC system is universal but some means-tested benefits	Main LTC public scheme is means-tested but some universal benefits	Access is means-tested for all benefits
Australia, Austria, Finland, France, Italy, Japan, Malta, Netherlands, Republic of Korea, Scotland, Slovenia, Spain, Sweden	France	Croatia, England^b^, Wales	Chile, Costa Rica, Cyprus, India, Türkiye, USA

*Note.* LTC = Long-Term Care.

a The amount of benefits or co-payments may be means-tested, but all citizens who require care are covered at least partly by the system.

b Non-means-tested benefits: a small cash benefit (attendance allowance, ranging from £73.90 to £110.40 per week) and nursing care.

In 14 countries there was a national instrument to assess needs, but there are differences in how it is used. In some countries it only provides guidance and its use varies across territories and localities. In seven countries there is an algorithm that determines the amount or type of entitlement based on the needs assessed. In contrast, in 12 of the countries the decision is based on professional judgement. The experts note that even in countries with an algorithm, professional judgement still has an impact on the outcome of the assessments. In all countries the needs assessments consider functional ability (Activities of Daily Living and Instrumental Activities of Daily Living), and many countries include cognitive impairment. In England and Wales, instead of measuring “deficits”, the assessment focuses on the outcomes that support would enable the person to achieve. A few countries also include needs for support in maintaining autonomy (see table 2).

**Table 2:** Types of needs considered in the assessments.

	ADL/IADL	Cognitive impairment and dementia	Mobility impairments	Sensory impairments	Social isolation	Support for autonomy	Potential prevention/ rehabilitation	Mental health	Supervision and safety	Continence	Nursing needs
Australia	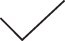	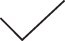	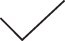	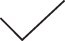	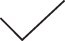	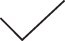	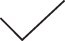	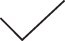	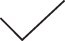	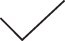	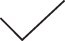
Austria	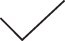	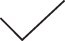	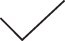	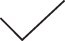						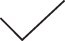	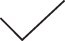
Chile	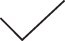										
Costa Rica	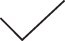										
Croatia	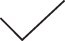	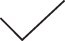			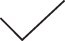	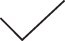			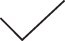		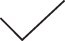
Cyprus	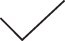		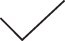	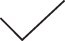		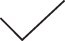					
England	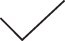	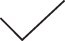	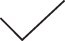	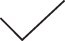	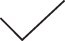	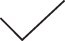	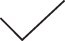	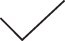	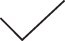	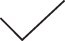	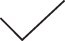
France	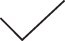		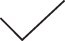	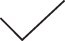						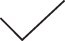	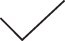
Finland	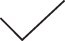	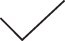	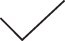	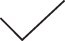	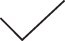	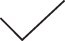	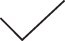	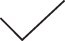	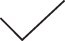	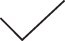	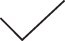
India											
Italy	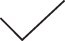		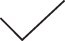	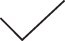						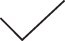	
Japan	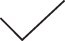	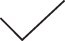	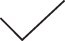	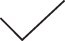	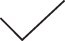	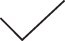	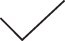	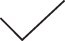	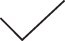	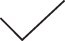	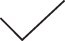
Malta	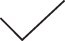	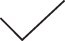	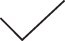	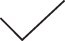	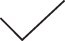	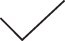	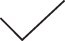	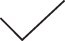	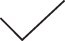	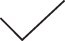	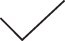
The Netherlands	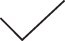	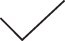	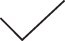	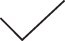	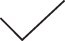	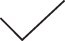	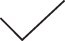	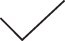	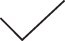	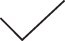	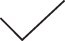
The Republic of Korea	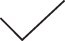	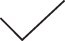	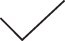				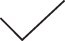	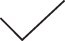	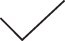	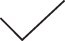	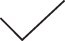
Scotland	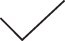	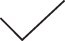	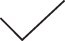								
Slovenia	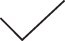	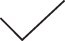	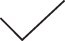		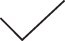	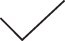		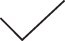	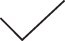	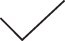	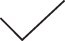
Spain	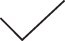	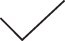	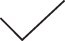							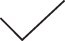	
Sweden	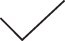	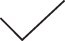	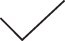	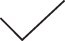	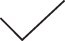	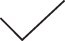	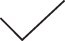	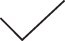	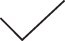	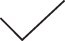	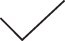
Türkiye	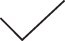	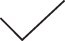	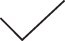	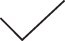				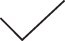			
USA (all states)	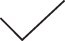										
USA (some states)	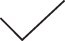	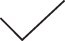	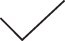	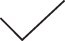	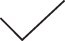	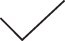	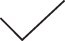	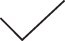	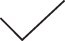	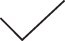	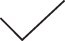
Wales	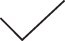	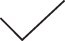	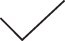	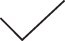	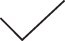	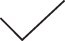	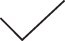	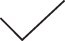	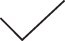	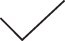	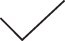

*Note.* ADL = Activities of Daily Living; IADL = Instrumental Activities of Daily Living.

Some systems cover only those with the most severe needs, whereas others take a more preventative approach, aiming to cover people early to postpone or reduce further functional dependency. Australia, Finland, Japan, Malta and Sweden cover all levels of care needs. Some countries that mostly cover those with moderate and severe needs are starting to offer some preventative care (table 3).

**Table 3:** Severity of needs covered.

All levels of care needs, including preventative services	Mostly moderate and severe needs with some preventative services	Moderate and severe needs only	Mostly severe needs only
Australia, Japan, Malta, Sweden	Chile, Cyprus, England, Finland, France, Netherlands, Republic of Korea, Scotland, Slovenia, Spain^c^, Wales	Austria, Costa Rica	Croatia^a^, India, Italy, Türkiye^a^, USA^b^

a There are exceptions for residential care as providers may refuse clients with high needs, this applies to public care homes in Türkiye

b At least severe needs, but States can cover less severe needs

c Limited availability

There are differences in coverage according to whether people have access to care from family or neighbours. The systems in Chile, Croatia, Cyprus and India target people without carers while other systems offer fewer benefits or lower priority to people with carers (Austria, England, USA). In Cyprus, India, Italy, Slovenia and Türkiye, offspring are financially liable for the care and support of their parents.

In some countries family and other kin carers are covered, while in others the beneficiary, in principle, is only the person with care needs, although services and benefits may indirectly support their family carers, as in Cyprus, France, India, Italy, Japan, Republic of Korea, Slovenia, Spain and the USA.

#### WHAT is covered?

In almost all countries there is public coverage for some form of cash benefits, home and community-based care and residential care, with the exception of India, where there are no nationwide systems. The relative importance and role of these different care approaches vary enormously. In some countries residential care is used mostly for people with low care needs who require support for social reasons (e.g. in Türkiye public care homes only admit people with low care needs and in Croatia many care homes avoid admitting people with complex needs), whereas in other countries residential care is aimed at those with the highest needs.

The role of cash benefits varies enormously: some systems do not have them (Australia, Finland, France, India, Japan, and Sweden), in some countries they consist of relatively small amounts linked to disability pensions, whereas in Austria and Italy, cash benefits are the main form of public coverage.

Most systems include some preventative care and/or rehabilitation, aids and housing adaptations, technology such as alarms and assisted housing (table 4). However, these are often considered part of the health or housing systems.

**Table 4:** Breadth of coverage of the public LTC systems.

	Preventative care/rehabilitation	Day care used for preventative purposes	Aids and adaptations	Technology such as alarms	Assisted housing
Yes	Australia, Austria^a^, Chile, Cyprus, England, Finland, France, India^b^, Italy^a^, Japan, Malta, Netherlands, Republic of Korea, Scotland^b^, Slovenia^b^, Spain, Sweden, USA^a^, Wales	Chile, Croatia, Finland, Japan, Netherlands, Spain^b^, Türkiye, USA	Australia, Austria, Chile, Croatia, Cyprus^b^, England, Finland, France, India^b^, Italy, Japan, Malta, Netherlands, Republic of Korea, Scotland, Slovenia^b^, Spain^a^, Sweden, Türkiye, USA^a^, Wales	Australia, Austria, Costa Rica, England, Finland, France, Italy^a^, Japan, Malta, Netherlands, Republic of Korea, Scotland^a^, Slovenia^b^, Spain, Sweden, Türkiye^b^, USA^a^, Wales	Australia^a^, Austria, Chile, Croatia, Cyprus^b^, England, Finland, France, Italy^a^, Malta, Netherlands, Republic of Korea, Scotland, Slovenia^b^, Spain^a^, Türkiye^b^, USA^a^, Wales

*Note.* LTC = Long-Term Care.

a Varies by locality, region or state

b Limited availability

**Table 5:** Coverage of services and benefits for family and other kin carers.

Direct coverage: respite care services	Direct coverage: counselling and peer support	Direct coverage: carer training	Direct cover: carer cash benefit	Direct cover: social security benefits	Direct cover: through a contract	Varies by State/region
Australia, Austria, Chile, Costa Rica, Croatia, Cyprus, England, Finland, France, Japan, Malta, Netherlands, Republic of Korea, Scotland, Slovenia, Sweden, Wales	Netherlands, Sweden	Australia, Austria*, Chile, Costa Rica, Malta, Netherlands^a^, Slovenia	Australia^b^, Austria^b^, Chile, Croatia, Cyprus, England, France, Malta^b^, Netherlands, Republic of Korea^c^, Scotland, Slovenia^b^, Türkiye, Wales	Austria^a^, Croatia, England, Netherlands, Spain	Austria^a^, Finland^a^, Netherlands, Slovenia^b^	Italy, Netherlands^a^, USA

a Varies by locality, region or state

b For carers of people with severe need who cannot work full time due to caring responsibilities

c Highly restrictive

Respite care is the most frequent support for family and other kin carers (although in many countries access is difficult), this can be in-home, day care, or short-term residential stays. Other carer supports include cash benefits and, in a few countries, family carers can be formally employed. Some countries offer carer leave, paid (Austria, Australia, Italy, Scotland, and some states in the USA) and unpaid (England, France, Japan, Malta, Slovenia, Spain, some states in the USA and Wales).

#### HOW much is covered?

The depth of coverage in a system is determined by the share of the costs that are met through out-of-pocket payments (OOP) by the people who are covered. In some countries with very strict means-tests to access public care there are no co-payments (for example Chile and Cyprus). In contrast, countries such as Australia with very broad coverage in terms of which needs are covered and the range of services, have substantial co-payments in place.

There are many ways in which OOP can be collected. In the countries in this study, we have found all of these: a fixed percentage of the cost of a service, a fixed percentage of someone’s income (or pension), the public system only covering a part of the cost of the service and the user needing to provide the remainder.

Where OOP are means-tested, some countries consider only income, whereas others also include assets, which may include housing (often considered only for residential care).

Many countries limit OOP by determining a minimum personal allowance that is exempted. In Australia there are also annual and lifetime caps placing limits on the total amount a person must pay for certain government-subsidised aged care fees to protect individuals from excessive care costs.

Rules regarding OOP often vary for different types of services and benefits, particularly for residential care compared to home and community-based care, where the costs linked to accommodation and certain services tend to be considered separately from the costs of care.

### Part two: Is public coverage of LTC expanding or contracting?

Considering the changes in coverage since 2021, we should note that not all the changes are due to reforms, as policy inaction impacts coverage too, e.g. if funding for LTC has not kept pace with demand, there may be increases in waiting lists or local commissioners may ration services by tightening eligibility or offering less intensity.

#### Changes in needs testing

In four countries needs tests became less stringent: in Australia and Malta to facilitate preventative approaches to care and, in France and Republic of Korea, to improve access for people living with dementia. Increased budgetary constraints faced by sub-national territories in England and Finland led to more stringent applications of needs tests, see table 6.

**Table 6:** Changes in needs tests since 2021.

More stringent (increased severity requirement)	Less stringent (decreased severity requirement)	No change
England^a^, Finland^a^, Slovenia, Türkiye	France^b^, Malta, Republic of Korea^b^	Australia, Austria, Chile, Costa Rica, Croatia, Cyprus, India, Italy, Japan, Netherlands, Scotland, Spain, Sweden, USA, Wales

a Stricter implementation of eligibility criteria

b Improved sensitivity to include people with dementia

#### Changes in means-testing

In three countries means-tests became more stringent (table 7). In contrast, Cyprus broadened eligibility by extending benefits to low-income pensioners and Malta eliminated means-testing for disability assistance. Italy introduced a temporary means-tested bonus. The USA state of California, experimented with phasing out an asset test between 2022 and 2025, but reinstated it in 2026. USA enacted a new law in July 2025 (the One Big Beautiful Bill Act, OBBBA) that will reduce maximum exemptions for primary home equity from 2028.

**Table 7:** Changes in means tests to access care since 2021.

More stringent	Less stringent	No change
Australia, Costa Rica, England^a^, Netherlands, Türkiye	Cyprus, Italy^b^, Malta	Chile, Costa Rica, Croatia, Cyprus, Finland, India, Japan, Republic of Korea, Scotland, Slovenia, Spain^c^, Sweden, USA^c^, Wales

a Through not uprating the means-tests to keep up with rises in incomes

b Temporary means-tested bonus

c Varies by locality, region or state

#### Changes to waiting times for assessments or services

Australia and Malta implemented reforms aiming to reduce waiting lists. Spain is currently debating a reform that aims to facilitate the assessment process and reduce waiting times. In Austria the expansion of services has not kept up with demand, leading to increased waiting lists and in England waiting lists for assessments grew during the COVID pandemic but are now starting to fall again (see table 8).

**Table 8:** Changes in waiting times for assessments or services since 2021.

Decreased waiting times (may include measures aiming to reduce waiting times even if there is no data on whether this has been successful)	Increased waiting times	No evidence of change
Australia, Malta, Netherlands	Austria, England	Chile, Costa Rica, Croatia, Cyprus, Finland, France, Italy, Japan, Republic of Korea, Spain^a^, Sweden, Türkiye, USA^b^, Wales

a A planned reform may change this

b Varies by locality, region or state

#### Changes in co- payments

Reforms in Australia increased co-payments for new entrants into the system, so that individuals with financial capacity pay a greater share of care costs. In the USA, the OBBBA increased cost-sharing for certain people enrolled in Medicaid from 2028 and reduced funding for Medicaid may result in future cost-cutting measures. In contrast, the new LTC Insurance Act in Slovenia eliminated co-payments for services provided under the Act, although this may be reviewed by 2028 (see table 9).

**Table 9:** Changes in co-payments since 2021.

Higher co-payments	Less stringent	No change	Unclear
Australia, Netherlands, Republic of Korea^a^, USA	Slovenia	Austria, Chile, Costa Rica, Croatia, Cyprus, England, France, India, Italy, Japan, Malta, Scotland, Spain^a^, Sweden, Wales	Finland

a A planned reform may change this

#### Favouring access to services with lower costs

Many countries have adopted policy changes that favour home and community-based care over residential care. Expansions and improvements in home and community-based care have been reported in 9 of the countries, and reductions in the availability of residential care in two. In Spain reforms to improve community-based care are in discussion, see table 10.

**Table 10:** Policies that favour home and community-based care since 2021.

Expanding and improving home and community-based care	Reduction of availability of residential care	No change
Australia, Austria, Croatia, Finland, France, Malta, Netherlands, Republic of Korea, Sweden, Türkiye	Finland, Sweden	Chile, Costa Rica, Cyprus, England, India, Italy^a^, Japan, Scotland, Slovenia, Spain^a^, USA, Wales

a There are legislative proposals to increase support for home and community-based care

#### Favouring preventative services to reduce or delay need for more intensive care

Australia, France, Japan, Malta, Slovenia and Sweden have introduced (or continued to implement) changes to improve access to preventative care and reablement, through the LTC system, e.g. day care, home-based care and technology. Another approach is to address this from the health system (in Australia, Chile, England, Malta, Republic of Korea, Türkiye and Wales).

#### Changes to services or benefits offered

There have been increases in the range of services or benefits available in Australia, Austria, Chile, Finland, India, Italy, Malta and Slovenia. Some of the new services or benefits target family and kin carers, such as a carers gateway to centralise support for carers in Australia, a carers allowance in Karnataka (India), financial support for carers in Austria and Malta, a national carers registry in Chile and a short break scheme in Wales (see table 11).

**Table 11:** Introduction or reduction of services or benefits since 2021.

Expansion of the range of services or benefits	Expansion of support for carers	Reducing services or benefits	No change
Australia, Austria, Finland, Italy^a^, Malta, Netherlands, Slovenia	Australia, Austria, Chile, India^b^, Malta, Republic of Korea		Costa Rica, Croatia, Cyprus, England, France, India, Japan, Republic of Korea, Scotland, Spain, Sweden, Türkiye, USA, Wales

a Temporary benefit

b In at least one State

#### Changing the intensity of services available or the value of benefits

Countries that are adopting preventative and community-first strategies, such as Australia, Republic of Korea and Slovenia, have expanded the intensity of home care. There have been increases in the value of benefits in Italy (limited to people with low incomes) and Malta. In contrast, the intensity of care seems to be decreasing in Finland and France.

#### Changes in the quality of care, including staff working conditions and training requirements

Cyprus, France, Japan, Malta, Republic of Korea, Spain, Türkiye and the USA reported changes aiming to improve quality of care, such as incentives to providers or new quality requirements. Many countries adopted measures to improve pay and working conditions in the sector and make training more accessible to people working in the sector.

We observed two contrasting trends: some countries increased staff to client ratios or requirements for staff training (often due to deficiencies observed in the pandemic, e.g. Australia, Korea, Spain and Malta). In other countries, worsened workforce shortages have resulted in temporary relaxations of staff ratios and training requirements (e.g. Finland and Sweden) as well as measures to make it easier to recruit people from third countries to work in LTC (e.g. Austria, Japan, Slovenia and, temporarily, England).

### Recent and expected reforms

While some countries have adopted major reforms since 2021 that are starting to impact the coverage of the risk of LTC (e.g. Australia (Watson & Dunsmore, 2025) and Malta, in others, reforms have not yet translated into visible changes in coverage, or the process of implementation has not yet begun, e.g. in Finland, France and Slovenia (Forma & Leinonen, 2024; Oung et al., 2025; Potočnik T et al., 2025). In Italy a major reform was adopted but its implementation has been postponed (Gubert & Perobelli, 2025) and similarly in Scotland, plans for a new National Care Service have been delayed.

Many countries adopted smaller reforms with measures to strengthen systems, e.g. in Austria three reform packages between 2022 and 2024 aimed to improve working conditions for formal carers, provide financial support for training, increase support for family carers, and addressed specific challenges of live-in care (Famira-Mühlberger & Österle, 2024).

Sweden has not had any new national reforms after 2021, partly reflecting the local nature of the responsibility of LTC, in practice, municipalities have mostly reduced coverage (especially for residential care) and emphasized care at home, and have high expectations of the potential for digital technology (Johansson, 2023).

There are a few countries where more reforms are planned or expected, e.g. Austria and England have established Commissions to prepare reform plans, Croatia, Cyprus and Malta are working on major reforms (Kantaris, 2025) and Spain has started the parliamentary process for a new reform.

## Discussion

This study highlights the complexity of the mechanisms determining the breadth and depth of coverage provided by public LTC systems internationally, in terms of the number of policy decisions involved in designing a system, and the variety of options countries have adopted.

The extent of coverage depends both on whether the system is universal as opposed to means-tested and on how the eligible needs and population are defined. Needs assessments can expand or reduce the numbers of people covered, depending on the types of needs considered and the severity required before a person is considered eligible. Whether and how family and kin carers are covered also affects the extent of coverage of the system.

Between 2021 and end of 2025 we observe a trend towards increasing breadth of coverage in terms of who is covered, with some counties expanding the types of needs covered to include dementia and cognitive impairment or including people with lower levels of needs to enable access to preventative services. Many countries are also expanding the types of care covered by the LTC system, increasing availability of home and community-based care and support for family carers.

[W]e observe a trend towards increasing breadth of coverage in terms of who is covered, with some counties expanding the types of needs covered to include dementia and cognitive impairment or including people with lower levels of needs to enable access to preventative services.

Many countries are also expanding the types of care covered by the LTC system, increasing availability of home and community-based care and support for family carers.

In terms of depth of coverage, we observe changes in the co-payment’s rules in both directions: some countries are making them more stringent so that wealthier people contribute more, whereas the means-tests in others have become less stringent.

The approach adopted here, mapping the mechanisms and policies to a set of policy questions does not allow for an evaluation of whether these policies are meeting their objectives, as that would require comparative quantitative data that is not currently available. What this approach allows is the examination of the congruence between the mechanisms and policies in place and the declared policy objectives. For example, where stated aims to embrace prevention are not well matched by highly restrictive needs assessments.

A limitation of our approach is that it requires that countries have at least some LTC schemes with rules about who is entitled, to what and how much. This makes it difficult to characterise countries such as India, which does not yet have a nationwide formal public system (although some states are developing policies) (Rajagopalan & Askandha, 2025).

Different political and policy traditions and economic circumstances impact countries’ ability to undertake and implement reforms, despite having strong awareness of the need to do so. While in most countries policy changes require major reforms to make any changes, Japan and Republic of Korea have built-in systems to review and update their system every few years (revisions of the fee-schedule in Japan and the Basic Plans for LTC in Korea) (Ping & Oshio, 2023). This approach enables these countries to update their systems regularly to respond to demographic, social and economic changes.

Even once a reform has been adopted, it may not be implemented, as seen in Italy and in England (Gubert & Perobelli, 2025; Needham & Burn, 2025). And even after the implementation has begun, it may take years for the consequences of the reforms to be observable. With some exceptions, major LTC reforms take time to be adopted and implemented (Götze & Rothgang, 2014; Potočnik T et al., 2025).

## Supplementary Material

prag006_Supplementary_Data
