# Supplementary material for: Public coverage of Long-Term Care in the post-COVID period: strengthening systems vs cost-containment
Source: Public Policy Aging Rep. 2026 May 14;36(1):14–23. doi: 10.1093/ppar/prag006 (PMC13240997; doi:10.1093/ppar/prag006)
Supplement: prag006_Supplementary_Data [file prag006_supplementary_data.pdf]

Public coverage of Long-Term Care in the post-COVID period: strengthening systems vs cost-containment

Corresponding author:

Adelina Comas-Herrera (a.comas@lse.ac.uk, Care Policy and Evaluation Centre, London School of Economics and Political Science

Name	Email	ORCID	Affiliation
Adelina Comas-Herrera, MSc	a.comas@lse.ac.uk	0000-0002-9860-9062	Care Policy and Evaluation Centre, London School of Economics and Political Science, London, United Kingdom
Nazak Salehi, MSc.	n.salehi@lse.ac.uk	0009-0004-6531-8498	Care Policy and Evaluation Centre, London School of Economics and Political Science, London, United Kingdom
Alexander Chaverri-Carvajal, PhD	achaverri@ced.uab.es	0000-0002-2203-2607	Centre d'Estudis Demogràfics, Universitat Autònoma de Barcelona, Bellaterra, Spain
Mari Aaltonen, PhD	mari.s.aaltonen@thl.fi	0000-0002-6620-4968	Department of Public Health and Welfare, Finnish Institute for Health and Welfare (THL), Helsinki, Finland
Başak Akkan, PhD	basak.akkan@bilgi.edu.tr	0000-0002-2387-5023	International Relations Department, Istanbul Bilgi University, Istanbul, Türkiye
Simon Bottery, BSc	s.bottery@kingsfund.org.uk		Policy Directorate, The King's Fund, London, United Kingdom
Jorge Browne, MD, MSc	jabrowne@uc.cl	0000-0001-6586-7084	Department of Public Health, Pontificia Universidad Católica de Chile, Santiago, Chile
Maria Cheshire-Allen, PhD	m.cheshire-allen@swansea.ac.uk	0000-0002-6026-6201	School of Health and Social Care, Swansea University, Swansea, Wales, United Kingdom
Margaret Dunham, PhD	m.dunham@napier.ac.uk	0000-0002-7244-1476	School of Health and Social Care, Edinburgh Napier University, Edinburgh, Scotland, United Kingdom
Moira Dunsmore, PhD	Moira.Dunsmore@sydney.edu.au	0000-0001-6394-2781	Sydney Nursing School, The University of Sydney, Sydney, New South Wales, Australia
Cansu Erdoğan Cengiz, MA	cansu.erdogan@uni-bielefeld.de	0009-0006-2192-8176	Faculty of Sociology, Bielefeld University, Bielefeld, North Rhine-Westphalia, Germany
Ulrike Famira-Mühlberger, PhD	Ulrike.Famira-Muehlberger@wifo.ac.at	0009-0003-8373-8608	Health Economics and Health Policy Unit, Austrian Institute of Economic Research (WIFO), Vienna, Austria
Maria Aurora Fenech, PhD	maria-aurora.fenech@um.edu.mt	0000-0002-9550-2729	Department of Health Services Management, University of Malta, Msida, Malta
Leena Forma, PhD	leena.forma@tuni.fi	0000-0002-8656-3722	Unit of Health Sciences, Tampere University, Tampere, Finland;<br>Department of Health and Social Management, University of Eastern Finland, Kuopio, Finland
Maya Fransz-Myers, MSL	franszmy@usc.edu		Center for Economic and Social Research, University of Southern California, Los Angeles, California, USA
Vlad Grigoras, PhD	vgrigoras@worldbank.org		Social Protection and Jobs Global Practice, World Bank, Washington, DC, USA
Ester Gubert, PhD	ester.gubert@unitn.it	0009-0005-5068-3836	Department of Sociology and Social Research, University of Trento, Trento, Italy
Ali Hamandi, MD PhD	ahamandi@worldbank.org	0000-0001-8069-564X	Health, Nutrition, and Population Global Practice, World Bank, Washington, DC, USA
Valentina Hlebec, PhD	valentina.hlebec@fdv.uni-lj.si	0000-0002-3691-7959	Faculty of Social Sciences, University of Ljubljana, Ljubljana, Slovenia
Maša Filipovič Hrast, PhD	masa.filipovic@fdv.uni-lj.si	0000-0003-4816-1164	Faculty of Social Sciences, University of Ljubljana, Ljubljana, Slovenia
Lennarth Johansson, PhD	svenlennart.johansson@ju.se	0009-0002-9246-4189	School of Health and Welfare, Jönköping University, Jönköping, Sweden
Marios Kantaris, PhD	marioskantaris@healthresearch.cy	0000-0003-4704-2649	Health Services and Social Policy Research Centre, Cyprus University of Technology, Limassol, Cyprus
Hongsoo Kim, PhD	hk65@snu.ac.kr	0000-0002-5539-7653	Department of Public Health Sciences, Graduate School of Public Health & Artificial Intelligence Institute, Seoul National University, Seoul, South Korea
David Knapp, PhD	dmknapp@usc.edu	0000-0002-9469-0692	Center for Economic and Social Research, University of Southern California, Los Angeles, California, USA
Henk Nies, PhD	h.l.g.r.nies@vu.nl	0000-0003-3897-206X	Department of Governance and Management in the Health and Care Sector, Vrije Universiteit Amsterdam, Amsterdam, Netherlands
Eleonora Perobelli, PhD	eleonora.perobelli@unibocconi.it	0000-0002-1578-005X	CERGAS, SDA Bocconi School of Management, Bocconi University, Milan, Italy
Ruru Ping, PhD	ruru.ping@r.hit-u.ac.jp	0000-0001-7946-7866	Hitotsubashi Institute for Advanced Study, Hitotsubashi University, Tokyo, Japan
Tjaša Potočnik, MSc	tTjasa.potocnik@fdv.uni-lj.si	0009-0007-1475-1787	Faculty of Social Sciences, University of Ljubljana, Ljubljana, Slovenia
Jayeeta Rajagopalan, MSc	j.rajagopalan@lse.ac.uk	0000-0002-1442-9786	Care Policy and Evaluation Centre, London School of Economics and Political Science, London, United Kingdom
Roberta Sultana, PhD	rsult01@um.edu.mt	0009-0004-1902-865X	Department of Health Services Management, University of Malta, Msida, Malta
Loïc Trabut, PhD	Loic.trabut@ined.fr	0009-0009-0817-9897	Mobility, Trajectories and territories Unit, French National Institut for Demographic Studies (Ined), Aubervilliers, France
Cristina Vilaplana-Prieto, PhD	cvilaplana@um.es	0000-0001-7701-0934	Faculty of Economics and Business, University of Murcia, Murcia, Spain
Pablo Villalobos Dintrans, PhD	Pvillalobos.d@gmail.com	0000-0002-2236-6447	1. Escuela de Salud Pública, Facultad de Medicina y Ciencias de la Salud, Universidad Mayor, Santiago, Chile<br>2. Centro de Observación y Análisis de Datos en Salud (CADS), Universidad Mayor, Santiago, Chile
Diego Wachs, PhD	dwachs@worldbank.org	0000-0002-0692-8104	Social Protection and Jobs Global Practice, World Bank, Washington, DC, USA

Karen Watson, PhD	Karen.Watson@sydney.edu.au	0000-0003-4934-582X	Sydney Nursing School, The University of Sydney, Sydney, New South Wales, Australia
Raphael Wittenberg, MSc	r.wittenberg@lse.ac.uk	0000-0003-3096-2721	Care Policy and Evaluation Centre, London School of Economics and Political Science, London, United Kingdom
Joseba Zalakain, BSc.	jzalakain@siis.net		SIIS Research Centre, Fundación Eguía-Careaga, Donostia-San Sebastián, Basque Country, Spain

Appendix. Questionnaire used to collect data from each of the countries.

COUNTRY QUESTIONS:

Country name (if answering for a specific region within a country, please let us know]:

Contact person(s) name, affiliation and email address:

PART 1. COVERAGE

1. Who is covered?

1. **How does the LTC system define its beneficiaries:**
   - **1.1. Choices about the level of care needs at which people become eligible**
     - 1.1.1. Which needs are covered: Type of needs (i.e. ADL/IADL only, cognitive impairment and dementia, mental health, social isolation, supporting autonomy, etc
     - 1.1.2. Severity of needs: more preventative vs only severe needs are covered
   - **1.2. The age at which people are covered**
     - 1.2.1. Does the system cover people of all ages or are there different schemes for younger adults and older people with care needs
   - **1.3. Whether the system also covers the needs of unpaid carers**
     - 1.3.1. Are unpaid carers beneficiaries? If yes:
     - 1.3.2. Are unpaid carers direct beneficiaries? (i.e. they are given services and/or benefits to meet their own needs as carers)
     - 1.3.3. Are unpaid carers indirect beneficiaries? (i.e. some services/benefits provided to the person in need of care are meant to support unpaid carers as well)
   - **1.4. Does the system offer coverage according to care needs only, or does it restrict public funding to people below a means test or who do not have access to family care**
     - 1.4.1. Does eligibility for services depend on income and assets? If so which sources of income and/or types of assets are included?
     - 1.4.2. Does eligibility depend on having access to unpaid care?
   - **1.5. How are needs measured?**
     - 1.5.1. Is there a national instrument for needs assessments?
     - 1.5.2. Which institution/government level/professional groups carry out the assessments?
   - **1.6. How is eligibility established?**
     - 1.6.1. Is there are fixed eligibility threshold for different types and quantities of benefits set at national or local levels, or is it based on professional judgement?

2. Which services/benefits are covered?

2.1. **Does the system cover in the same way all of these services**: home-based, community-based (e.g. day care) and residential care?

2.2. **Does the system offer cash benefits?** How can they be used?

2.3. Does the system include "preventative" care and rehabilitation?

2.4. Does it cover aids and housing adaptation?

2.5. Does it cover technology such as alarms?

2.6. Is there public funding for housing with support (or assisted housing)[1]?

2.7. Is eligibility determined in the same way for all of these services/benefits, or are there different systems/approaches?

2.8. What types of support for unpaid carers are available:

2.8.1. respite/formal care (non-residential), residential respite?

2.8.2. financial support: salary, pension and social insurance contributions?

2.8.3. Paid or unpaid carer leave?

3. How much of the costs of care are covered?

3.1. Are co-payments determined at national or local level?

3.2. Are there the same co-payments for home, community-based and residential care services?

3.3. Are co-payments means-tested?

3.3.1. Are co-payments designed as flat rates (i.e. the same percentage for all), or do they increase with the level of income?

3.3.2. Are income and/or assets are taken into consideration?

3.4. If the benefits provided do not cover the whole cost of a service, are there social assistance or other means of supporting people who cannot afford to buy all the care they need?

PART 2. Cost-containment:

In this section we seek to explore whether your country has adopted cost-containment policies since the Covid-19 pandemic (e.g. since 2021). In this part we build on a paper by Gori and Luppi (2024)[2]. They identify demand side and supply side policies to contain costs of care.

The question here is whether your country has adopted any of these policies since 2021, it may be that some countries have adopted them in the opposite direction:

1. Demand side:

1.1. Tightening/expanding eligibility criteria by changing:

1.1.1. Needs testing (increasing the level of needs required to be entitled to a benefit (or the opposite)

---

[1] See definitions for assisted housing here: https://www.who.int/europe/publications/i/item/WHO-EURO-2024-10363-50135-75510

[2] https://www.cambridge.org/core/journals/ageing-and-society/article/costcontainment-longterm-care-policies-for-older-people-across-the-organisation-for-economic-cooperation-and-development-oecd-a-scoping-review/470A5966A6292AAEAC303F669058293A#article

1.1.2. Means testing (introducing or removing means-test, increasing/decreasing the generosity of the tests, for access to services

1.1.3. Carer sighting (making easier/harder for people who have a family carer to access publicly funded care)

1.2. Reducing/Increasing care responsiveness by influencing the timing, i.e. increasing/reducing waiting times for services.

1.3. Introducing/removing or increasing/reducing co-payments

2. Supply side:

2.1. Changing the mix of services available:

2.1.1. Favouring access to services with lower costs (e.g. home over residential care, cash over in-kind benefits)

2.1.2. Favouring preventative services to reduce the need for more intensive care later on (for example reablement services, preventative services such as day care, health checks, social prescribing, etc.)

2.2. Changing the intensity and quality of services:

2.2.1. Directly: decrease/increase in service intensity (for example average number of hours of home care, number of weekly visits to day centres, etc)

2.2.2. Indirectly: reducing/increasing the requirement for staff training (or language requirements), accepting worse/better working conditions, changes in the levels of need at which there is an entitlement to publicly funded care, (de-)professionalisation of care (changes in staff training requirements, working conditions, public purchasers paying more/less for services.
